# Difficulties in accessing health services among the elderly in the city of São Paulo-Brazil

**DOI:** 10.1371/journal.pone.0268519

**Published:** 2022-05-19

**Authors:** Elaine Cristina Tôrres Oliveira, Marília Cristina Prado Louvison, Doralice Severo da Cruz Teixeira, Tarciana Nobre de Menezes, Tereza Etsuko da Costa Rosa, Yeda Aparecida de Oliveira Duarte

**Affiliations:** 1 Faculty of Public Health, University of São Paulo (USP), São Paulo, São Paulo, Brazil; 2 Department of Policy, Management and Health, Faculty of Public Health, University of São Paulo (USP), São Paulo, São Paulo, Brazil; 3 Faculty of Public Health, Center for Studies on Aging, University of São Paulo (USP), São Paulo, São Paulo, Brazil; 4 Department of Physiotherapy, State University of Paraíba (UEPB), Campina Grande, Paraíba, Brazil; 5 Health Institute of the São Paulo State, Department of Health, São Paulo, São Paulo, Brazil; 6 Coordinator of the Health, Well-Being and Aging Study (SABE), School of Nursing, Faculty of Public Health, University of São Paulo (USP), São Paulo, São Paulo, Brazil; National Research Centre of Egypt, EGYPT

## Abstract

To identify difficulties in accessing health services by the elderly in the city of São Paulo/Brazil and the contributory factors that reflect inequalities. This is a cross-sectional study that used data from the Health, Well-being and Aging Study (SABE). The population is composed of elderly ≥ 60 years old, of both sexes, living in the urban area of São Paulo. For this analysis, we used data from the 2015 cohort of the SABE study, containing a sample of 1,221 individuals. The proportions of access difficulty and, through logistic regression, the associated factors were verified, based on Andersen’s Behavioral Model, which considers factors of predisposition, enabling and need as individual determinants of access to health care. It was observed that 37.0% of the elderly reported difficulty accessing health services when they needed it. This difficulty was greatest among females (42.3%), aged 60 to 69 years (40.9%), black race/color (58.8%), illiterate (44.5%), single/separated/divorced (44.3%), with income slower than one salary minimum (46.8%), without health insurance (51.9%), with poor/very poor self-assessment of health (54.7%), with multimorbidity (40.1%), frail (47.2%) and among those who used polypharmacy (40.8%). After multivariate analysis, in the final model, there was a positive association between difficulty of access and predisposing factors (female gender, age group 60 to 69 years, black race/color, illiterate), enabling factors (possession of health insurance) and need factors (regular and poor/very poor self-assessment of health and pre-fragility and frailty condition). The presence of difficulty in access associated with predisposing, enabling and need factors reflect the existence of inequalities caused by barriers that point to weaknesses in the organization of services. The identification of these barriers that hinder access highlights important points that can have an impact on the equity and resolution of care.

## Introduction

Population aging, considered one of the global demographic megatrends, has triggered discussions on the international scene with a view to developing measures that ensure healthy living and promoting the well-being of all, at all ages [1]. These discussions have been based on the need to include prospective policies in global agendas that can absorb and deal with the needs and specificities of an aging world [1, 2].

Demographic projections indicate that the elderly population will become increasingly numerous in the world, which will, in addition to opportunities, face challenges to the contemporary context. According to United Nations estimates, in absolute numbers, the world will rise from approximately 703 million elderly (65 years or older) in 2019 to one and a half billion elderly by 2050, an increase of around 120% in just 30 years. Analyses on territorial distribution indicate that two-thirds of these elderly people will live in low- and middle-income countries by 2050, a situation that raises concerns due to fiscal and political pressures that will be faced over the years in relation to health, retirement and social protection systems [1].

Brazil, like the other countries in Latin America and the Caribbean, has experienced the accelerated increase in the number of elderly in its population, a condition that is an imminent challenge to be faced in view of its occurrence in a scenario marked by inequalities [3]. According to projections, in 2050, 28% of the Brazilian population will be composed of individuals aged 60 years or more, a proportion approximately 2.7 times higher when compared to the proportion of the 2010 demographic census [4].

In addition to demographic concerns, aging implies social and economic repercussions if these years are overdue by disabilities and dependence [5]. It is a crucial point for sustainable development to ensure that aging occurs in a healthy way, however the analyses carried out on life expectancy and the number of years expected to live in health have revealed worrying data [6].

Indicators about the patterns and trends of healthy life years have revealed that the accelerated increase in life expectancy in populations has not resulted, at the same speed, in an increase in healthy life expectancy [7]. This finding has suggested the occurrence of an absolute expansion of morbidity associated with longevity, a condition that leads to the hypothesis that the demand for health services will grow due to population aging [8] and will require health systems to reorganize care to increase the supply of services [9].

Analyses on the demand for health services reveal that the elderly, when compared to other population groups, use more health services, present a higher frequency of hospitalizations, in addition to greater care needs due to greater multimorbidity [9]. This condition reinforces the need to ensure access to services in order to maintain continuous and permanent monitoring of health conditions [10] and face the difficulties of aging [5].

Considering this need for care in the face of aging, it is important that health systems identify which elements are involved in obtaining access to health services. It is understood that access is a complex multidimensional phenomenon, which constitutes a category of analysis of health systems because it refers to the act/opportunity that individuals have to enter the system and have continuity of care from the need approach [11].

Access is configured as an important characteristic of study on the use of health services and its analysis can reveal the existence of factors that cause resistance or difficulty in the use of services among individuals, essential information in the scope of organization, financing and delivery of health services to the population [12]. By identifying the elements related to access to health services by the elderly, it is possible to expand the analysis of the dimension of the use of services and verify the main factors linked to barriers and inequalities in access to health services [13].

The search for determining factors and the production of inequalities in access and use of services among the elderly has been presented as a theme of interest for several studies due to its contribution to the development of policies promoting equity. Both in national studies [14–18] and as international [19–23], some factors have been associated with difficulty in the use and access of health services among the elderly. Among the factors observed, demographic (gender [17, 22, 23] and age [18–20]) and socioeconomic (income [14, 19–22] and health insurance [14,19–21]), social structure (race/color [16, 22] and schooling [14, 18, 23]) and health conditions (perception of health [15, 19, 21] and multimorbidity [17, 20, 21]) have been important determinants for accessing health care.

Explanatory theoretical models have sought to define and measure the factors that structure and/or influence access to and use of health services. Among the proposed models, Andersen’s Behavioral Model has been of great applicability in the use studies [24] and considers that individual determinants are related to access to health services and can express from the dominant factors, the existence or not of inequalities [11].

Among the individual determinants, the model proposes that predisposing (demographic and social structure), enabling (facilitating resources) and health needs factors are related to access to health services. Based on the analysis of these individual determinants, the model considers that access is equitable when demographic and need variables account for most of the utilization of services. When factors related to social structure and enabling resources predominate in determining the use of services, there is evidence of inequalities in access to health services [11].

Although universal health systems, as in the case of Brazil, have as a premise the organization of access to health services based on needs, differences in the implementation of health actions and services may incur in inequalities [25]. Studies that sought to identify inequalities in access to health services in Brazil found that income, education and possession of health insurance are some determining of access in the country [14, 26–28]. Regarding the elderly, besides these variables, an association with gender, the presence of diseases and self- perception of health was also identified, which refers to the need for the organization of services taking into account the specificities of this population [29].

It is important to highlight that the use of health services is often used as an access proxy, making it a measure without being directly observed. This analysis of access ends up demonstrating the inequalities only in relation to those who used the services, in greater or lesser volume, not considering the individuals who may have sought the service and could not perform care, nor those who, despite achieving, had many barriers in carrying it out. A study conducted in the city of São Paulo with a previous cohort [29], which used the look at access from the use of health services, identified factors associated with inequalities in the use and access of health services, but without specifically analyzing the difficulties, which brings to the present analysis an innovation and new findings. Therefore, it is important that studies seek to verify the existence of difficulties in order to be able to access health services and identify the factors involved in the construction of these inequalities, in addition to behavioral analyses of use.

Aiming at the organization of access to health services, the execution of studies that seek to understand the difficulties perceived by the elderly during the search for health services, with consequent identification of associated factors contributes to the construction of paths that facilitate the entry of individuals into the system and the reduction of inequities.

Given the complex structure of access to health services in Brazil [30], as well as the accelerated aging process of the country with an estimated peak for the coming decades [31], it is important to identify which factors would be contributing to the existence of difficulties in accessing health among the elderly and, eventually, the existence of inequalities. In this sense, the present study aims to identify difficulties in accessing health services by the elderly in the city of São Paulo, SP, Brazil, as well as the contributory factors that reflect inequalities.

## Materials and methods

This research is part of the Health, Well-Being and Aging Study (Saúde, Bem-Estar e Envelhecimento—SABE) which aims to assess the health status of older people and broaden the dialogue between public health research and the study of aging [32]. It was initially conceived by the Pan American Health Organization (PAHO) as a multicenter survey to be carried out seven urban centers in Latin America and the Caribbean. The choice of the participating countries was based, on one hand, on the combination of a good representation of the aging stages in the region, and on the other hand, on the possibility of obtaining the material and human resources required for the study development [31]. In Brazil, the city of São Paulo was indicated as the research site, which, despite not having the largest proportion of elderly people in the country, represented and still represents the city with the largest absolute number of elderly people and the greatest local diversity resulting from immigration and internal migration [33].

**Fig 1:**
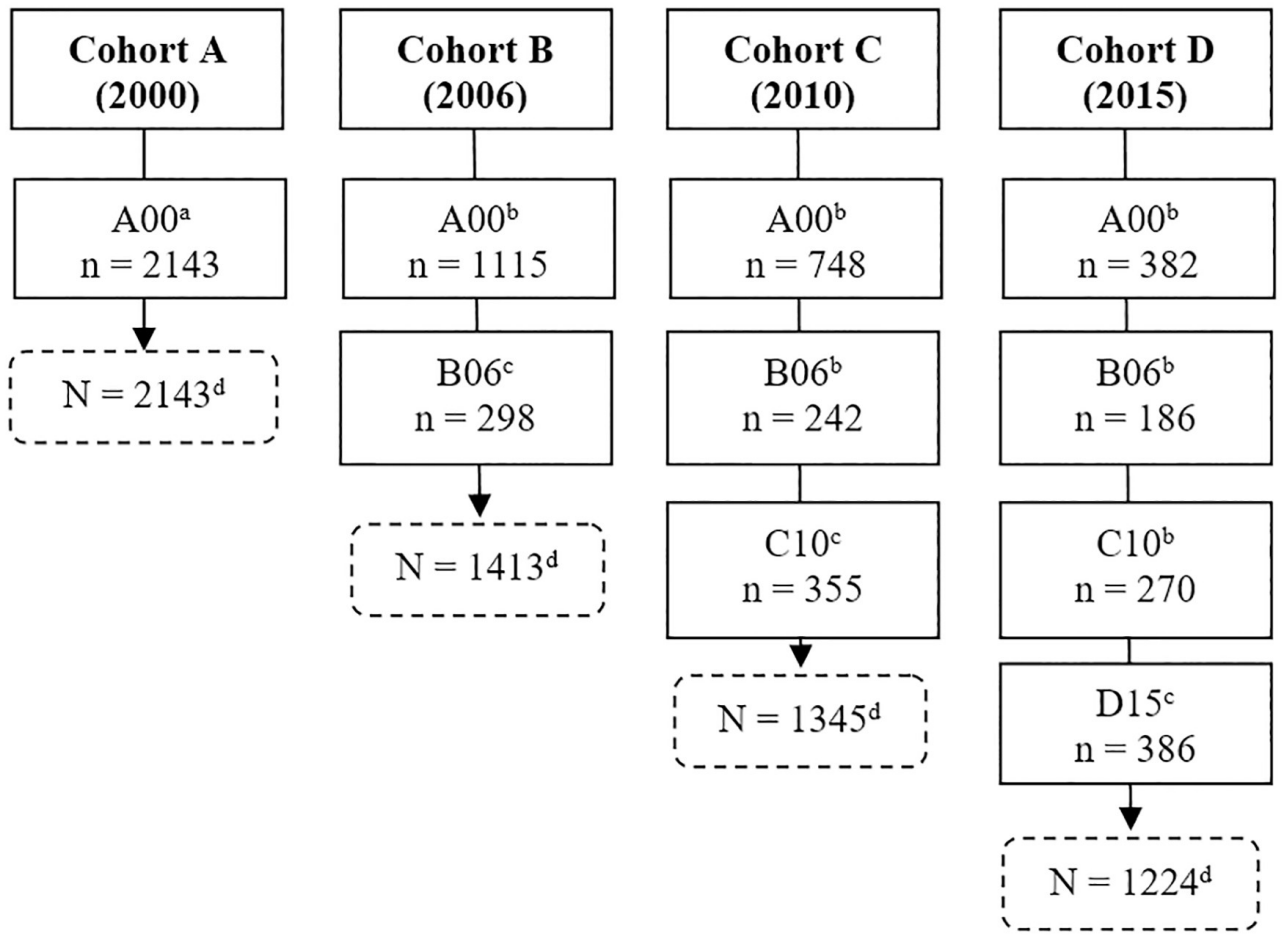
Sample distribution of the SABE Study, years 2000, 2006, 2010 and 2015. São Paulo-Brazil. ^a^ Primary sample. ^b^ Reinterviewed elderly. ^c^ Elderly people aged 60 to 64 included. ^d^ Number of elderly in each cohort.

The population of interest of the SABE Study is composed of individuals aged 60 years or older, non-institutionalized, of both sexes, living in urban areas, selected by cluster sampling [32]. In Brazil, the SABE Study became a longitudinal study of multiple cohorts, and the individuals were interviewed for the first time in 2000, revisited and reevaluated in the years 2006, 2010 and 2015. In each new period, survivors of previous samples were interviewed and individuals aged between 60 and 64 years were added to compensate for aging in the population base and maintain representativeness in all age groups [34]. The number of individuals interviewed and added to each cohort is described in Fig 1.

In each cohort, the individuals were weighted relative to the effects of the sampling design, which made the samples representative for the municipality population in terms of the age group and corresponding year [34]. The sampling design included census tracts as primary analysis units and randomly selected households as secondary analysis units. Details about the sampling design are described in other publications [31–33].

For the present study, which has a cross-sectional approach, we used information from the 2015 cohort. It is important to note that the SABE Study is a study of multiple cohorts that allows a cross-sectional cut from the survivors of the 2000, 2006 and 2010 cohorts and a subsample of individuals aged 60 to 64 years participating in the 2015 cohort. Given this possibility, the population of this study corresponded to individuals from the 2000 (382), 2006 (186), 2010 (270) and 2015 (386) wave, totaling 1,224 respondents (Fig 1). The elderly who did not know or did not answer the questionnaire about the difficulty of access to health services appropriate to their needs, and those with missing information about this variable (n = 03) were excluded from this study, resulting in a final sample of 1,221 individuals.

Data collection was carried out by interviewers trained by the research coordinators, who used maps marked with the respective streets, block faces, and households to be visited, in addition to the research instruments (forms). The return of the forms by the interviewers was marked by an accounting of the fieldwork control instruments and an initial review of their completion and consistency. Any doubts were clarified with the interviewers, if necessary, new contacts were made with the elderly, by phone or in person, in the search of clarification. After criticism, the forms were coded and then typed [31].

For the purpose of analysis and study of possible associations, the difficulty of access to health services when needed was considered a dependent variable. The information about the difficulty of access was verified using the following question: " *In relation to the service that you use most often*, *do you have any difficulty in access/use health services when you need them*?". Among the answer options (yes, no, don’t know and/or didn’t answer), those identified as yes or no were considered for analysis. We chose to use the difficulty of access perceived by the elderly as a category of analysis in order to value the perception of the individual about the barriers faced in meeting health care needs.

The independent variables were organized based on predisposing, enabling and need characteristics related to the difficulty of access to health services among the elderly. They were selected from Andersen’s conceptual structure that considers individual determinants of access to health care, based on a behavioral model of use of services, capable of expressing inequalities [11]. Based on this, this study used independent variables for the analysis of access to health services, information regarding predisposing characteristics (demographic and social structure); enabling (socioeconomic conditions) and need (perceived and evaluated health conditions). On demographic and social structure information, the gender (male, female), age group (60 to 69 years, 70 to 79 years, 80 years and more), race/color (white, black and others), years of study (illiterate, 1 to 3 years, 4 to 7 years, 8 to 11 years, were included in the observation, 12 years or more) and marital status (married/cohabitation, single/separated/divorced, widowed) of the elderly participants.

For the analysis of socioeconomic variables, information on income (less than 1 minimum wage, between 1 and 2 minimum wages and more than 2 minimum wages) and possession of health insurance (yes, no) were included. Income information was obtained through a national reference that is characterized by minimum wage numbers received per month [35]. In Brazil, the minimum wage represents the minimum amount that an employed person must receive from his employer for his/her monthly work. The base value is set in law, nationally unified and capable of meeting the basic vital needs of the worker and his family [36]. It is important to emphasize that it is constitutionally established that no benefit that replaces the contribution salary or income from work will have a monthly value lower than the minimum wage, a condition that applies to income received by elderly people in the country. The answers related to income are due to the following question: "*In total*, *approximately*, *how much do you receive per month*?". The minimum wage corresponding to the time of data collection and which was taken as a reference was 880.00 reais per month (about 225 U.S. dollars). The information about the possession of health insurance (health insurance/insurance) was obtained by questioning "*Do you have any health insurance* (health insurance/insurance) *besides the Unified Health System*?", and the answer given by the elderly was analyzed in a dichotomous way.

Regarding the health conditions of the elderly participants, information regarding self- assessment of health (very good/good, regular, bad/very bad), the presence or not of multimorbidity (0 to 1 chronic disease, 2 or more chronic diseases), frailty syndrome (non-fragile, pre-fragile, fragile) and the use or not of polypharmacy (0 to 4 medications, 5 or more medications) were considered for the analysis and study of possible associations. The construction of the variable self-assessment of health occurred from the question: " *Would you say that your health is…*", and the answers were codified as very good, good, regular, bad and very bad. For statistical purposes, the classification of the self-rated health of each elderly person was redefined into categories.

For the analysis of multimorbidity, the number of chronic non-communicable diseases reported by the research participants was considered. The report of chronic diseases was obtained by questioning "*Has a doctor or nurse ever told you that you have*…", and the answers are limited to eight chronic diseases (systemic arterial hypertension, diabetes, chronic lung disease, heart disease, stroke, joint disease, osteoporosis and cancer). The presence of multimorbidity was considered the report of two or more chronic non-communicable diseases [37].

For the analysis of frailty syndrome, we used the construct proposed by Fried et al. [38] that analyzes five components of a phenotype and classifies the elderly as non-frail (the one who does not have phenotype components), pre-fragile (the one with one or two components of the phenotype) and fragile (the one with three or more components of the phenotype). This classification of the elderly as to the presence or not of frailty syndrome occurred by verifying the following components, as described in the publication by Duarte et al. [37]:

- Unintentional weight loss: weight loss equal to or greater than three kilograms in the last 12 months, without diet, was verified by positive reporting.
- Fatigue reported: analyzed from the frequency, in the last week, of great effort to perform routine activities and the feeling of not being able to take things forward (started something, but could not finish). The answers to both questions were classified as: seldom or no time (less than 1 day), some part of the time (one to two days), a moderate part of the time (three to four days), or all time.
- Decrease in strength: verified from grip measured by dynamometer in the dominant hand, adjusted for sex and body mass index (BMI). In this component, the elderly with positive scores were considered those who were located in the lowest quintile of the strength distribution, according to sex and BMI quartile.
- Gait speed decrease: according to gender and height, from the three-meter walking test present in the Short Physical Performance Battery Assessing Lower Extremity Function [39]. The analysis of this component was verified from the stratification in quintile of gait speed distribution, according to gender and median height value of the elderly.
- Low level of physical activity: analyzed by checking the weekly energy expenditure in kilocalories, adjusted by gender, according to the performance of physical activities and exercises reported by the elderly. To identify this component, we used the short version (translated) of the International Physical Activity Questionnaire (IPAQ) which analyzes walking, moderate physical activity, vigorous physical activity and leisure activities, home and/or work [40]. The weekly calorie expenditure according to the activities performed was verified by measuring the total metabolic equivalents (METs) spent during the period, multiplied by the result of dividing the elderly’s weight by 60, and presented in quintiles according to gender [41]. The elderly belonging to the lowest quintile of caloric expenditure according to sex scored positively in this component.

Finally, the analysis of the use of polypharmacy occurred through the following questions: "*Could you show me the medications (medicines) you are currently taking (using)*?" and "*Could you tell me the name of the medications (medicines) you are taking (using)*?". All drugs shown and/or reported were recorded, being considered for analysis purposes, elderly people using polypharmacy those who used five or more medications [42].

### Statistical analysis

Initially, the proportions of elderly with difficulty in accessing health services according to predisposing, enabling and need variables were verified. Because the analysis occurred in complex samples, the Pearson test with Rao-Scott correction was used, which considers the sample weights with estimates for population weighting. To identify the factors associated with the difficulty of access to health services, univariate logistic regression analyses were performed, with calculation of crude odds ratio (OR) and confidence interval (CI) of 95%. For the elaboration of the final model, multivariate logistic regression analysis was used, with the forward stepwise inclusion method, for the calculation of the adjusted OR. Three adjusted models were created, the first (Model 1) containing the predisposing variables, the second (Model 2) containing the predisposing and enabling variables and the final model (Model 3) with all previous variables followed by the need variables.

In the final model, variables with significance levels greater than 5% were excluded. The statistical information was obtained with the aid of Stata^®^ software, version 11, using the survey module due to the characteristics of the complex design of the sample.

### Ethical aspects

All the elderly signed the Informed Consent Form after verbal and written explanations regarding the study. The SABE Study was submitted and approved by the Research Ethics Committee of the School of Public Health of the University of São Paulo, with its 2015 cohort approved opinion under CAAE number 47683115.4.0000.5421.

## Results

The sample of this study was composed of 1,221 elderly (65.20% of women). The participants’ age ranged from 60 to 101 years, with a mean of 71.4 years (SD = 9.14). It was found that the proportion of elderly who had difficulty in accessing health services upon need was 37.03%. Table 1 shows the distribution of the elderly according to the difficulty of access, predisposing, enabling and need characteristics. It is observed that the difficulty of accessing health services was greater among elderly females (42.26%), age 60 to 69 years (40.94%), black (58.82%), illiterate (44.55%), single/separated/divorced (44.33%), with an income of less than one minimum wage (46.85%) and without health insurance (51.86%).

**Table 1:** Distribution of the elderly according to the difficulty of access to health services, predisposing, enabling and health need characteristics. SABE Study, São Paulo, Brazil, 2015.

Variables	N (%)	Difficulty of access to health services	
		% (95% CI^a^)	p-value
**Total**	**1,221 (100)**	**37.03 (32.96, 41.29)**	**-**
***Predisposing characteristics***			
**Sex**			
Male	425 (34.8)	30.27(24.80, 36.36)	<0.001^b^
Female	796 (65.2)	42.26(37.94, 46.71)	
**Age group**			
60 to 69 years	675 (55.3)	40.94(35.41, 46.72)	0.0497^b^
70 to 79 years	309 (25.3)	34.78(27.41, 42.96)	
80 years or more	237 (19.4)	27.59(20.49, 36.03)	
**Race/Color**			
White	627 (51.3)	33.14(28.01, 38.70)	<0.001^b^
Black	87 (7.1)	58.82(47.13, 69.60)	
Others	492 (40.3)	38.68(33.57, 44.05)	
**Years of study**			
Illiterate	169 (13.8)	44.55(35.46, 54.02)	0.001^b^
1 to 3 years	226 (18.5)	42.67(35.43, 50.23)	
4 to 7 years	452 (37.0)	37.00(31.30, 43.10)	
8 to 11 years	245 (20.1)	36.92(29.97, 44.46)	
12 years or more	126 (10.3)	19.90(12.91, 29.39)	
**Marital status**			
Married/cohabitation	608 (49.8)	35.01(29.77, 40.64)	0.0732
Single/separated/divorced	220 (18.0)	44.33(37.14, 51.75)	
Widowed	393 (32.2)	36.71(31.47, 42.28)	
***Enabling characteristics***			
**Income** ^c^			
Less than 1 minimum wage	48 (3.9)	46.85(30.42, 63.99)	0.0623
Between 1 and 2 minimum wages	557 (45.6)	40.51(35.40, 45.84)	
More than 2 minimum wages	616 (50.5)	33.38(28.11, 30.09)	
**Health insurance**			
Yes	564 (46.2)	19.87(16.02, 24.38)	<0.001^b^
No	657 (53.8)	51.86(46.54, 57.14)	
***Need characteristics***			
**Self-rated health**			
Very good/good	576 (47.2)	29.27(23.90, 35.29)	<0.001^b^
Average	529 (43.3)	42.95(37.45, 48.63)	
Bad/very bad	86 (7.0)	54.72(41.66, 67.16)	
**Multimorbidity**			
0 to 1 chronic disease	468 (38.3)	32.37(26.52, 38.83)	0.0184^b^
2 or more chronic diseases	753 (61.7)	40.14(35.78, 44.66)	
**Frailty syndrome**			
Not fragile	389 (31.9)	30.85(25.14, 37.20)	0.0098^b^
Pre-fragile	663 (54.3)	38.68(33.54, 44.08)	
Fragile	167 (13.8)	47.25(37.71, 57.00)	
**Polypharmacy**			
0 to 4 medications	739 (60.5)	34.61(29.76, 39.81)	0.0467^b^
5 or more medications	482 (39.5)	40.85(35.60, 46.31)	

^a^ 95% CI: 95% Confidence Interval.

^b^ Statistically significant association (p<0.05).

^c^ Value of the minimum wage numbers received per month at the time of data collection taken as a reference was 880.00 Brazilian reais (about 225 U.S. dollars).

In the analysis of health conditions, it was possible to verify that the difficulty in accessing health services was greater among the elderly with a self-assessment of bad or very bad health (54.72%), with multimorbidity (40.14%), in frailty condition (47.25%) and who used polypharmacy (40.85%). Among the variables assessed, only marital status and income showed no statistically significant association with difficulty of access (p > 0.05).

Table 2 shows the values of the univariate analysis corresponding to the difficulty of access to health services and associated factors, as well as the values of the crude odds ratios. From the results it is possible to identify that among the predisposing characteristics the following were considered factors associated with the difficulty of access to health services: females gender when compared to males; age group 80 years or older when compared to 60 to 69 years, race/color black when compared to white, 12 years or more of schooling when compared to illiterate, and marital status single, separated or divorced when compared to married.

**Table 2:** Logistic regression in relation to the outcome difficulty in accessing health services by the elderly and associated factors. SABE Study, São Paulo, Brazil, 2015.

Variables		Model 1	Model 2	Model 3
	Crude OR (95% CI^a^)	Adjusted OR (95% CI^a^)	Adjusted OR (95% CI^a^)	Adjusted OR (95% CI^a^)
***Predisposing characteristics***				
**Sex**				
Male	1	1	1	1
Female	1.686[1.289, 2.205]^b^	1.648[1.229, 2.210]^b^	1.891[1.402, 2.549]^b^	1.863[1.375, 2.524]^b^
**Age group**				
60 to 69 years	1	1	1	1
70 to 79 years	0.769[0.507, 1.166]	0.671[0.431, 1.043]	0.785[0.493, 1.250]	0.663[0.405, 1.084]
80 years or more	0.549[0.347, 0.869]^b^	0.446[0.271, 0.733]^b^	0.651[0.398, 1.065]	0.477[0.284, 0.801]^b^
**Race/Color**				
White	1	1	1	1
Black	2.882[1.711, 4.852]^b^	2.384[1.384, 4.106]^b^	1.927[1.113, 3.336]^b^	2.005[1.126, 3.567]^b^
Others	1.272[0.952, 1.699]	1.078[0.797, 1.459]	0.974[0.704, 1.349]	0.993[0.721, 1.368]
**Years of study**				
Illiterate	1	1	1	1
1 to 3 years	0.926[0.585, 1.464]	0.776[0.476, 1.263]	0.811[0.487, 1.351]	0.849[0.516, 1.395]
4 to 7 years	0.731[0.474, 1.127]	0.611[0.400, 0.935]^b^	0.680[0.435, 1.063]	0.732[0.467, 1.146]
8 to 11 years	0.728[0.436, 1.216]	0.539[0.318, 0.913]^b^	0.688[0.409, 1.157]	0.750[0.443, 1.269]
12 years or more	0.309[0.164, 0.582]^b^	0.245[0.131, 0.458]^b^	0.464[0.249, 0.865]^b^	0.546[0.299, 0.996]^b^
**Marital status**				
Married/cohabitation	1	1	1	1
Single/separated/divorced	1.478[1.069, 2.042]^b^	1.229[0.876, 1.724]	1.059[0.728, 1.539]	1.073[0.737, 1.563]
Widowed	1.076[0.796, 1.454]	0.951[0.669, 1.351]	0.827[0.585, 1.170]	0.821[0.581, 1.160]
***Enabling characteristics***				
**Income** ^b^				
Less than 1 minimum wage	1		1	1
Between 1 and 2 minimum wages	0.772[0.373, 1.598]		0.991[0.496, 1.977]	0.995[0.455, 2.005]
More than 2 minimum wages	0.568[0.287, 1.122]		1.191[0.616, 2.301]	1.200[0.592, 2.432]
**Health insurance**				
Yes	1		1	1
No	4.344[3.221, 5.858]^b^		4.012[2.972, 5.415]^b^	3.992[2.892, 5.508]^b^
***Need characteristics***				
**Self-rated health**				
Very good/good	1			1
Average	1.821[1.325, 2.505]^b^			1.418[1.022, 1.968]^b^
Bad/very bad	2.914[1.632, 5.204]^b^			1.943[1.038, 3.638]^b^
**Multimorbidity**				
0 to 1 chronic disease	1			1
2 or more chronic diseases	1.400[1.058, 1.852]^b^			1.028[0.690, 1.531]
**Frailty syndrome**				
Not fragile	1			1
Pre-fragile	1.414[1.042, 1.918]^b^			1.520[1.096, 2.107]^b^
Fragile	2.008[1.198, 3.364]^b^			2.245[1.317, 3.826]^b^
**Polypharmacy**				
0 to 4 medications	1			1
5 or more medications	1.304[1.009, 1.695]			1.300[0.886, 1.908]

^a^ 95% CI: 95% Confidence Interval.

^b^ Statistically significant association (p<0.05).

^c^ Value of the minimum wage numbers received per month at the time of data collection taken as a reference was 880.00 Brazilian reais (about 225 U.S. dollars).

In the analysis of the enabling characteristics, it was verified that the absence of health insurance among the elderly studied was associated with difficulty in accessing health services. Regarding the characteristics of need, it was verified that negative perception of health, presence of multimorbidity and conditions of frailty were associated with the presence of the outcome (Table 2).

After multivariate analysis and presentation of the final model (Model 3), it was observed that sex, age group, race/color, years of study, health insurance, self- assessment of health and frailty syndrome (p<0.05) remained associated with difficulty of access (Table 2). It was found that female seniors and black race/color seniors had increased chances of 1.86 [1.37, 2.52] and 2.05 [1.13, 3.57], respectively, of facing difficulties in accessing health services when they needed it. Also on the predisposing characteristics, we observed that elderly people aged 60 to 69 years and illiterate had increased chances of facing difficulties in accessing health services when compared to their peers (0.47 [0.29, 0.80] and 0.55 [0.29, 0.99], respectively).

Regarding the enabling characteristics, it was observed that the possession of health insurance presented a significant relationship with access to health services in the population studied (Table 2). It was found that elderly who reported not having health insurance had 3.99 [2.89, 5.50] more chances of difficulty accessing health services due to need when compared to the elderly who reported having health insurance.

Finally, among the characteristics of need analyzed and that remained statistically associated in the final model, it was observed that elderly people with a perception of regular, bad and very bad health presented, respectively, 1.41 [1.02, 1.96] and 1.94 [1.03, 3.63] more chances of difficulty in accessing health services when compared to those who evaluated their health as very good and good. Similarly, elderly who were in pre-frailty and frailty condition presented increased chances of difficulty in accessing health services when compared to non-fragile ones (1.52 [1.09, 2.10] and 2.24 [1.31, 3.82], respectively).

## Discussion

A considerable proportion of the elderly in this study reported difficulty in accessing health services through need was verified among the elderly in this study. This result was similar to that found by a population study conducted in the city of Montes Claros (MG), which identified a prevalence of 33.0% of difficulty in using the main health service among the elderly, when necessary [15]. The existence of a considerable number of elderly people who identified difficulty in accessing health care is worrisome and needs to be analyzed, considering the importance of facilitating the use of health services for this population group as the imminent risks of worsening pathological processes that may occur by non-monitoring and monitoring of the health conditions of these individuals are considered. In this sense, it becomes a crucial point to organize the health care of the elderly that favors the reduction of possible complications and hospitalizations due to avoidable conditions, as well as contributing to measures that promote healthy aging.

Based on the assumption that the existence of equity in access contributes to the reduction of imminent health risks, this study sought to verify which factors constituted barriers to access and care of needs among the elderly studied. It was verified that predisposing, enabling and need characteristics were significantly associated with difficulties in accessing health services. The relationship observed between some of the characteristics associated with difficulty of access may promote the reproduction of social inequities [11].

It was observed that elderly females had greater difficulty accessing health services when compared to males, a result that does not corroborate the findings of other studies that sought to perform a gender analysis [23, 43, 44]. A population survey in Zhejiang Province, China, which sought to examine access to health services among the elderly, identified that women living in urban areas had lower chances of difficulty in hospitalization due to need when compared to men [43]. In the same way, a study conducted in Turkey that sought to investigate the use of health services and satisfaction of the elderly found that women had a lower number of unmet health needs when compared to men (p<0.01) [23].

This analysis, based on the gender approach, has been evidenced in studies on the use and access to health services [21, 23, 43–45] with a view to understanding the user profile of the services, as well as the organization of the care provided. It is important to highlight that this gender difference has the involvement of a social and cultural component that influences the use of health services, including among the elderly [46].

Considering a social and cultural scenario in health favorable to women [46], a potential explanation for the identification of a higher chances for difficult in accessing to the gender in this study may be related to the occurrence of greater demand for health services among the elderly women studied, which causes greater probability of facing barriers of access. A study that analyzed the access and use of health services in men and women in Brazil, identified a small gender difference in favor of men among people who sought care in health services and were not attended [47]. The identification of the aspects involved in the reported difficulty is fundamental for understanding the reasons related, thus configuring sex/gender as an important element of analysis in the face of the (re)organization of the care offered by the services.

Another characteristic identified in this study that evidenced the existence of barriers to access to health services concerns race/color. It was verified that elderly black people presented greater difficulties in accessing services due to need when compared to whites, a result that corroborates findings from other studies [16, 45, 48, 49]. A population study that sought to examine and compare the rates of use and access of health services among racial/ethnic groups in the United States identified that black individuals presented worse performance in access to health care when compared to whites, a condition independent of gender [48].

The difficulty of accessing and use health services from a racial approach was also evidenced by a study conducted in the city of São Paulo that found that white elderly could perform a greater number of tests requested by health professionals when compared to brown and black elderly (p = 0.011) [16]. The identification of differences in access from a racial perspective raises concerns regarding the existence of cultural barriers that interfere in care and promote disparities in obtaining health care.

It is interesting to note, based on the final model analyzed, that black race/color, regardless of the level of education and possession of health insurance, maintains a strong association with difficulty of access, indicating that there was no result bias by confounding effect. In this sense, it is necessary that structural inequalities become the focus of intervention so that health care offered according to the needs of individuals can be guaranteed.

The analyses on predisposing characteristics also showed that aspects related to age group and schooling acted as factors for the difficulty of access to health services among the elderly. It was observed that younger elderly people presented increased chances of difficulty in accessing health services when compared to younger ones, a result similar to that found by other studies [23, 44, 50]. A study conducted in Ghana, Africa, on equity in the use of health services by individuals aged 50 years and more identified an increasing association between the use of services and the increase in age, to the point that elderly people aged 80 years and older presented an increased chances of 1.69 [1.20, 2.37] of use of outpatient services when compared to those aged 50 to 59 years [44].

This identification of a lower chances of difficulty in accessing health services from increasing age reinforces the need to understand about the events associated with the phenomenon. This may be due either to the organization of care due to a higher occurrence of pathological conditions that required care, or even due to the older elderly, because they are survivors, present a state of health that allows a lower demand for services and facing less difficulties, or a greater resilience about the perception of access difficulties. The organization of health services and systems that promote greater opportunity for care due to age becomes important, considering the possibility of monitoring the processes related to senescence and early action on factors related to senility.

Another aspect that was configured as a protective factor for access to health services in the elderly studied was the fact that individuals with higher schooling had a lower chances of reporting access difficulties when compared to those without schooling, a result that corroborated other studies [14, 21, 23, 44, 51]. A study conducted with a stratified and representative sample of the Ghanaian population identified a positive and statistically significant association between access to outpatient services and schooling, so that elderly with higher education and high school had 1.60 [1.00,2.55] and 1.68 [1.27,2.23] respectively, higher probability of accessing outpatient care when compared to illiterate patients [44].

A potential explanation for the association between access to health services and years of study may be related to the fact that higher education can determine better jobs and income, besides offering greater opportunities for access to information. These conditions are favorable on the one hand, to the triggering of greater demand for health care, and on the other hand, the ease of access and use of health services by individuals [51]. However, the identification of a positive association between schooling and access to health care through need evidences the presence of inequalities that tend to benefit those who had greater opportunities for schooling.

When analyzing the enabling characteristics of access to health services, it was verified that the absence of health insurance presented the greatest contributing to the difficulty of accessing health services among the elderly in this study. This result is similar to the findings of other studies that sought to evaluate access to health services from the funding approach [14, 19, 20, 22, 52]. A study conducted in Indonesia based on national survey data to determine the factors associated with the use of outpatient services identified that elderly (60 years and older) who had health insurance had 1.38 [1.14,1.67] more chances to use outpatient services when compared to those without health insurance [21]. A result similar to that found in the United States where individuals who are not insured by health plans are more likely not to have medical consultations, tests or treatments when compared to those insured, because of the cost of services [22].

This relationship between financing and access to health services has been the object of discussion and constitutes a challenge to be faced, especially in the Brazilian context where health is a constitutional right [14]. Brazil presents a health system of peculiar format in relation to the other countries due to its three subsectors: a public, in which services are financed and provided by the State; a private, for-profit or non-profit, in which services are financed with public and private resources; and finally, a supplementary health subsector, represented by different types of private health plans and insurance policies, in addition to tax subsidies [30].

With a configuration that provides public and private participation in the financing and provision of health care [14], people are allowed to use all three subsectors in Brazil depending on the ease of access or their ability to pay [30]. However, considering that the social protection of health in Brazil comes from a national system of universal character that holds among its doctrinal principles the equity of care, it is worrying that the possession of health insurance determines access to services through need among the elderly studied. Improving access, implementation of policies to protect and refit the service network are urgent measures to reduce inequalities.

This study also sought to analyze within the scope of the necessity characteristics of the Andersen model [11], the relationship between the health conditions of the elderly and the difficulty of access to health services. It was observed that elderly with worse self-rated health status had greater difficulties in accessing the services when compared to those who rated their health status better, a result that was similar to that identified by other studies [15, 53].

A study conducted in the United States on timely access to medical care among non-institutionalized elderly identified that the probability of problems in accessing health services was higher among individuals who assessed their health as regular or precarious when compared to those who reported good, very good and excellent health [53]. Conversely, a study conducted in Indonesia found that elderly people who classified their health as regular and bad or very poor had 2.10 [1.49,2.95] and 2.22 [1.24,3.96], respectively, higher chances of using outpatient health services when compared to those who rated their health as optimal [21].

The finding that elderly people with worse self-rated of health status had greater difficulties in accessing services may be related to the occurrence of a higher demand for health care, which would lead to a greater probability of facing barriers of access. However, considering the importance of continuous and needs-focused monitoring, the identification of difficulty in access among those with worse health assessments raises concerns regarding the prevention of disabilities and dependence among individuals.

Also in the context of the analysis of health conditions and access to services, it was found that the elderly in this study in fragile condition presented greater possibilities of reporting difficulty of access when compared to non-frail ones, a result that corroborated the findings of another study conducted in Brazil [15]. A study that used the Edmonton Frail Scale to assess frailty in elderly residents in an urban area of a municipality in southeastern Brazil found that frail elderly had 1.35 [1.10,1.65] higher chances to have difficulty accessing health services through need when compared to non-frail ones [15].

It is important to understand the aspects involved with the identification that elderly people in a process of frailty or in a frailty condition experienced greater difficulties in accessing health services in this study. Among the possible explanations for this fact, it can be considered that a greater demand for care due to the state of health reverts in greater probability of experiencing difficulties in the use of services, or can also evidence that non-frail elderly have a lower demand for services, which has less repercussion in less experience of difficulty.

The results found by this study sought to verify difficulties in accessing health services by the elderly in in a Brazilian metropolis, as well as the contributory factors that reflect inequalities. However, as limitations of the study, the findings presented should be analyzed based on the cross-sectional characteristic of the research that does not allow the realization of causal inference. Other limitations attributed to the study refer to the memory bias in identifying the difficulty of access reported by the elderly when seeking services by necessity, the formulation of the question that may have a subjective bias in view of its relationship with the service of priority use by the elderly in the face of need and the measurement of the variables schooling, income and frailty that can influence different measures of effect. Despite these limitations, the methodology used in this study was sufficient to meet the objectives and the associations found are compatible with other studies conducted.

With the prolongation of longevity accompanied by increased morbidity, it is urgent that the various countries encourage and be prepared to ensure that the aging process occurs actively and healthily [3]. For this, it is necessary an intense relationship between health services and the elderly in order to optimize their health and increase the chances of enjoying a quality old age [15]. By understanding the access and use of health services as an important component in the aging process, this study made important notes on potential factors that can foster inequalities in access to health services by the elderly, which enables decision-making in view of the organization of health systems in order to consider the needs of individuals.

## Conclusion

In this study, a considerable proportion of elderly people who reported difficulty in accessing health services when to need, a condition that was associated with predisposing characteristics (gender, age group, race/color, schooling), enabling (health plan) and need (self-rated of health and frailty) were found. The identification of contributing factors for access difficulty reflects the existence of inequalities caused by barriers that point to weaknesses in the organization of services. The identification of these barriers that hinder access highlights important points that can have an impact on the equity and resolution of care.

The information found contributes to the knowledge about access to health services by the elderly and reveals the need for effective equity policies that consider the specificities of this population. The difficulties observed reveal inequalities in access, generated by social inequities, and require reorganization of care so that access reflects in meeting needs.

## Supporting information

S1 File Minimal data set. Set of data analysed for each individual include in this study.
(XLSX)
